# Endorsement of the CONSORT Statement by high impact factor medical journals: a survey of journal editors and journal 'Instructions to Authors'

**DOI:** 10.1186/1745-6215-9-20

**Published:** 2008-04-18

**Authors:** Sally Hopewell, Douglas G Altman, David Moher, Kenneth F Schulz

**Affiliations:** 1 Centre for Statistics in Medicine, University of Oxford, Oxford, UK; 2 Chalmers Research Group, Children's Hospital of Eastern Ontario Research Institute, Ottawa, Canada; Department of Epidemiology and Community Medicine, Faculty of Medicine, University of Ottawa, Canada; 3 Family Health International, P.O. Box 13950, Research Triangle Park, USA

## Abstract

**Background:**

The CONSORT Statement provides recommendations for reporting randomized controlled trials. We assessed the extent to which leading medical journals that publish reports of randomized trials incorporate the CONSORT recommendations into their journal and editorial processes.

**Methods:**

This article reports on two observational studies. Study 1: We examined the online version of 'Instructions to Authors' for 165 high impact factor medical journals and extracted all text mentioning the CONSORT Statement or CONSORT extension papers. Any mention of the International Committee of Medical Journal Editors (ICMJE) or clinical trial registration were also sought and extracted. Study 2: We surveyed the editor-in-chief, or editorial office, for each of the 165 journals about their journal's endorsement of CONSORT recommendations and its incorporation into their editorial and peer-review processes.

**Results:**

Study 1: Thirty-eight percent (62/165) of journals mentioned the CONSORT Statement in their online 'Instructions to Authors'; of these 37% (23/62) stated this was a requirement, 63% (39/62) were less clear in their recommendations. Very few journals mentioned the CONSORT extension papers. Journals that referred to CONSORT were more likely to refer to ICMJE guidelines (RR 2.16; 95% CI 1.51 to 3.08) and clinical trial registration (RR 3.67; 95% CI 2.36 to 5.71) than those journals which did not.

Study 2: Thirty-nine percent (64/165) of journals responded to the on-line survey, the majority were journal editors. Eighty-eight percent (50/57) of journals recommended authors comply with the CONSORT Statement; 62% (35/56) said they would require this. Forty-one percent (22/53) reported incorporating CONSORT into their peer-review process and 47% (25/53) into their editorial process. Eighty-one percent (47/58) reported including CONSORT in their 'Instructions to Authors' although there was some inconsistency when cross checking information on the journal's website. Sixty-nine percent (31/45) of journals recommended authors comply with the CONSORT extension for cluster trials, 60% (27/45) for harms and 42% (19/45) for non-inferiority and equivalence trials. Few journals mentioned these extensions in their 'Instructions to Authors'.

**Conclusion:**

Journals should be more explicit in their recommendations and expectations of authors regarding the CONSORT Statement and related CONSORT extensions papers.

## Background

It is over ten years since the CONSORT (Consolidated Standards of Reporting Trials) Statement was first published in 1996 [1] providing recommendations for reporting parallel group randomized controlled trials (RCTs). CONSORT provides recommendations for authors regarding how to prepare reports of trial findings, facilitating their complete and transparent reporting, and aiding their critical appraisal and interpretation. It has been endorsed by the World Association of Medical Editors (WAME), the International Committee of Medical Journal Editors (ICMJE), the Council of Science Editors (CSE), and well over 200 journals worldwide [2]. The revised CONSORT Statement of 2001 [3] has been translated into several languages and cited in journals over 1,300 times.

In addition to the CONSORT Statement, which provides recommendations for reporting parallel group randomized trials, extensions have been developed to give additional guidance for randomized trials with specific designs, data and interventions. These included the CONSORT extensions for cluster trials [4], non-inferiority and equivalence trials [5], herbal interventions [6], non-pharmacological interventions [7], abstracts [8] and harms [9].

Several studies have assessed the impact of using the CONSORT Statement to improve the reporting of RCTs in journal articles. Eight studies were summarized in a systematic review by Plint and colleagues [10], who concluded that journal adoption of the CONSORT Statement is associated with improved reporting of randomized trials although poor reporting remains common.

In 2003, Altman conducted a study of journal endorsement of the CONSORT Statement and found that seven years following its initial publication, and two years after its update, only 22% of 166 high impact factor journals provided any mention of CONSORT in their published 'Instructions to Authors' [2]. Four years have elapsed since this journal endorsement survey and we believe it is again timely to assess the impact of the CONSORT Statement and determine the extent to which journals that publish reports of randomized trials incorporate the CONSORT recommendations into their journal and editorial processes.

## Objectives

We set out to determine the extent to which leading medical journals incorporate the CONSORT Statement and related extension papers in their published 'Instructions to Authors'. We also sought to survey journal editors' opinions about endorsement of the CONSORT Statement and related CONSORT extension papers and their incorporation into the editorial and peer-review process.

## Methods

### Sample

Journals were selected using the strategy adopted by Altman [2]. Using citation impact factors (via ISI Web of Knowledge) for 2006, we identified the top five journals from each of the 33 medical specialties and the top 15 journals for general and internal medicine. Journals that did not publish clinical research (based on explicit statement or inspection of journal contents) were excluded and replaced by the next one on the list.

### Survey of journals' published 'Instructions to Authors'

For each of the journals in the sample, we examined the 'Instructions to Authors' published on the journal's website (searched between July and October 2007) and extracted all text mentioning CONSORT or CONSORT extension papers (cluster, non-inferiority and equivalence, herbal and harms). Any mention of the ICMJE's 'Uniform Requirements for Manuscripts Submitted to Biomedical Journals' or reference to clinical trial registration were also sought and extracted.

### Survey of journal editors

For each of the journals in the sample, we contacted via email the editor-in-chief, or editorial office if contact details for this person were not available (depending on the contact information included on the journal website), asking them to complete a short on-line survey (conducted between 6 August and 8 October 2007 using Survey Monkey). Two email reminders were sent to encourage editors to complete the on-line survey. The survey asked about their journal's endorsement of the CONSORT Statement, whether CONSORT is included in their 'Instructions to Authors', and if it is incorporated into their editorial and peer-review processes and, if so, how. Journal editors were also asked whether they recommended or required authors to comply with any of the CONSORT extension papers (cluster, non-inferiority and equivalence, herbal and harms). The on-line survey of journal editors' endorsement of CONSORT allows a direct comparison with what is reported in their journal's published 'Instructions to Authors' on their journal's website.

## Results

One hundred and eighty journals were identified, of which 15 were duplicates (i.e. listed in another medical speciality) and excluded; 165 journals were included in our sample.

### Survey of journals' published 'Instructions to Authors'

Of the 165 journals, 62 (38%) mentioned the CONSORT Statement in their 'Instructions to Authors' published on their journal's website. This is a relative increase of 73% from the survey in 2003 [2], when 36 (22%) of the 166 journals provided any mention of CONSORT. One-hundred and twenty-one journals were included in both the 2003 and 2007 samples; of these, 32 (26%) of 121 journals in 2003 provided any mention of CONSORT compared to 47 (39%) in 2007, a relative increase of 50%.

Twenty-three of the 62 journals that mentioned the CONSORT Statement in their 'Instructions to Authors' stated that this was a requirement of their journal (i.e. authors must conform to the CONSORT Statement) (Table 1). Of these, 17 stated that they required a completed CONSORT checklist to be submitted with the manuscript as a condition of the submission. The remaining 39 journals were less clear in their recommendations and included phrases such as authors "should consult the CONSORT guidelines" or "we encourage authors to follow the CONSORT Statement". Very few journals provided any mention of the CONSORT extension papers (cluster (n = 5), non-inferiority and equivalence (n = 1), herbal (n = 2) and harms (n = 3)).

**Table 1:** Mentioned in journals published 'Instructions to Authors'

	**N = 165 (%)**
**CONSORT Statement**	**62 (38%)**

Require	23
Recommend	39
Submit with checklist	17
Web address	46 *
2001 journal article	18
2001 exploratory article	1
Article citing 2001 article	2
1996 journal article (out-of-date)	6 **

**Cluster extension**	**5 (3%)**

Require	3
Recommend	2
Suitable reference	2

**Harms extension**	**3 (2%)**

Require	2
Recommend	1
Suitable reference	1

**Herbal extension**	**2 (1%)**

Recommend	2
Suitable reference	2

**Non-inferiority and equivalence extension**	**1 (1%)**

Recommend	1
Suitable reference	1

**ICMJE**	**69 (42%)**

Web address	48
Suitable reference (article > 2000)	3
Obsolete reference (article < 2000)	15
No reference	6

**Trial registration**	**61 (37%)**

Require	44
Recommend	17
Cites	23
Cites	9
Cites WHO International Clinical Trial Registry Platform	4
Cites a combination of the above	10

* Web address was misspelt (n = 2)

** Article citing the 1996 article (n = 1)

The majority of journals (n = 46) mentioning the CONSORT Statement gave the web address [11], while 18 referred to the latest version of the publication. Only one journal referred to the full explanatory publication [12], however, and six journals still referred to the out-of-date 1996 Statement [1].

Of the 165 journals, 69 (42%) referred to the ICMJE guidelines in their published 'Instructions to Authors'. This is a slight decrease from when this survey was last carried out in 2003, when 72 of the 166 (43%) journals referred to the ICMJE guidelines. The majority of journals (n = 48) referred to ICMJE the web address, including three which also referred to a recent publication. The remaining 15 journals cited an obsolete journal publication (i.e. published before 2000), while six included no reference. Journals that referred to CONSORT were much more likely to refer to the ICMJE guidelines (39/62; 63%) than those journals that did not refer to CONSORT (30/103; 29%) (relative risk 2.16; 95% confidence interval (CI) 1.51 to 3.08).

Sixty-one (37%) of the 165 journals mentioned clinical trial registration in their 'Instruction to Authors', of which 44 specifically stated that all recent clinical trials must be registered as a requirement of submission to that journal. A further 17 journals were less clear in their recommendations and, while they encouraged clinical trial registration, this was not a specific requirement. Cited sources of information about trial registration included the ICMJE web address (n = 23), ClinicalTrials.gov (n = 9) and the WHO International Clinical Trials Registry Platform (n = 4); the other 10 journals cited a combination of these sources. Again, journals that referred to CONSORT were much more likely to mention clinical trial registration (42/62; 68%) than those journals that did not refer to CONSORT (19/103; 18%) (relative risk 3.67; 95% CI 2.36 to 5.71).

Those journals which provided some mention of the CONSORT Statement in their 'Instructions to Authors' were also much more likely to include information about other reporting guidelines. These included QUOROM (13/165; 8%), MOOSE (7/165; 4%), REMARK (1/165; 1%), STROBE 4/165; 2%) and TREND (1/165; 1%); all mentioned CONSORT in their 'Instructions to Authors'.

### Survey of journal editors

Responses were received from 64 (39%) of the 165 journals to the on-line survey of journal editors about endorsement of the CONSORT Statement and related CONSORT extension papers (not all journals responded to each section of the survey). The majority of responders were journal editors (Table 2). Thirty-nine (61%) of the 64 responders reported that the CONSORT Statement was mentioned in the 'Instructions to Authors' on their journal's website.

**Table 2:** Survey of journals editors' endorsement of the CONSORT Statement

	N = 64* (%)
Person completing survey	
Editor in chief	18 (28%)
Managing editor	17 (27%)
Associate editor	7 (11%)
Editor	4 (6%)
Administrator	16 (25%)
Other (director)	2 (3%)

Journal recommends authors comply with the CONSORT Statement	50/57 (88%)
*Not applicable*	*2/57 (3%)*
Journal requires authors comply with the CONSORT Statement	35/56 (62%)
*Not applicable*	*2/56 (4%)*
Journal mentions the CONSORT Statement in its 'Instructions to Authors'	47/58 (81%)
*Not applicable*	*2/58 (3%)*
Journal incorporates the CONSORT Statement in its peer review process	22/53 (41%)
Journal incorporates the CONSORT Statement in its editorial process	25/53 (47%)

* Some responders did not complete all sections of the survey.

For 50 (50/57; 88%) journals, responders said that their journal recommended that authors comply with the CONSORT Statement; for 35 (35/56; 62%) journals this was a requirement (Table 2). Most respondents (47/58; 81%) also said that their journal mentioned the CONSORT Statement in its 'Instructions to Authors'. However, there is some inconsistency, as when subsequently cross checking the information provided on the journal's website under 'Instructions to Authors', 11 of the 47 journals provided no mention of the CONSORT Statement despite indicating in the on-line survey that they did.

Just under half of journals (22/53; 41%) said that they incorporated the CONSORT Statement into their peer-review process. A similar proportion of journals (25/53; 47%) said that they incorporated the CONSORT Statement into their editorial process. Examples of how this was achieved include "requiring authors to include a CONSORT flow diagram and completed checklist with their manuscript submission", "editors pre-screening and returning non compliant manuscripts directly to the authors", "including the CONSORT checklist for download on the peer review website alongside the submitted manuscript", "including information about the CONSORT Statement in the instructions for peer review", and "including the CONSORT Statement as part of the editorial checklist".

Journal editors were also asked about their journal's endorsement of extensions to the CONSORT Statement. Thirty-one (69%) journals said that their journal recommended authors comply with the CONSORT extension for cluster trials; 22 (49%) journals said that they would require this (Table 3). Twenty-seven (60%) said that their journal recommended authors comply with the CONSORT extension for harms and 18 (40%) said that they would require this. Nineteen (42%) journals said that their journal recommended authors comply with the CONSORT extension for non-inferiority and equivalence trials whereas 13 (29%) journals said that they would require this. The CONSORT extension for herbal interventions was not as widely endorsed as it was not applicable for some journals.

**Table 3:** Survey of journals editors' endorsement of extensions to the CONSORT Statement *

	**Yes**	**No**	**Not applicable**
**Journal recommends authors comply with the CONSORT**			
Cluster extension	31 (69%)	10 (22%)	4 (9%)
Harms extension	27 (60%)	11 (24%)	7 (16%)
Herbal extension	13 (29%)	10 (22%)	22 (49%)
Non-inferiority and equivalence extension	19 (42%)	12 (27%)	14 (31%)

**Journal requires authors comply with the CONSORT**			
Cluster extension	22 (49%)	16 (36%)	7 (15%)
Harms extension	18 (40%)	18 (40%)	9 (20%)
Herbal extension	12 (26%)	14 (30%)	20 (44%)
Non-inferiority and equivalence extension	13 (29%)	17 (39%)	14 (32%)

**Journal mentions CONSORT extension in its 'Instructions to Authors'**			
Cluster extension	12 (27%)	28 (62%)	5 (11%)
Harms extension	10 (22%)	29 (65%)	6 (13%)
Herbal extension	5 (12%)	24 (56%)	14 (32%)
Non-inferiority and equivalence extension	5 (12%)	28 (65%)	10 (23%)

* Some responders did not complete all sections of the survey.

Despite journal editors' responses about extensions to the CONSORT Statement, few actually mentioned these extensions in their 'Instructions to Authors' (Table 3).

## Discussion

The CONSORT Statement aims to improve the quality of reports of randomized trials. It is encouraging that over a third (38%) of high impact factor journals, assessed in our study, refer to the CONSORT Statement in their published 'Instruction to Authors'. This represents a relative increase of 73% since this study was last conducted in 2003 when only 22% of journals mentioned CONSORT in their 'Instruction to Authors' [2]. Some journals, however, still refer to the superseded 1996 version of the CONSORT Statement and we would encourage journals to keep their 'Instructions to Authors' up-to-date.

There is, however, still ambiguity in the wording of some journals' published 'Instructions to Authors' as to whether they require, or recommend, that authors comply with the CONSORT Statement [2]. We believe that journals should provide a clear message to their authors and recommend authors submit a completed CONSORT flow diagram and checklist as a requirement to submission in an endorsing journal. Only by endorsement of the CONSORT Statement by more journals, and greater editorials efforts to ensure that authors comply, can the quality of reporting of randomized trials published in leading journals be improved [10].

In September 2004, the members of the ICMJE published a joint editorial stating that they would only consider a trial for publication if it has been registered before the enrolment of the first patient [13]. In our study, over a third (37%) of journals required recent clinical trials to be registered as a requirement of submission to that journal. Disappointingly, a number of these journals did not provide the same endorsement of the CONSORT Statement despite recognising the importance of trial registration which, by itself, is not the best marker of trial quality.

It is also disappointing that so few journals endorse the recent extensions to the CONSORT Statement [4-6,9]. These extensions reflect a considerable amount of work and were developed to improve the reporting and transparency of trials using specific designs and types of data. Other CONSORT extensions have very recently been published [7,8] and others are being developed. We would recommend that journals reference these extensions in their 'Instructions to Authors' thereby helping authors wanting to improve the reporting of their randomized trials.

One obvious limitation of our study is that, despite several electronic reminders, there was a poor response rate (39%) to the on-line survey of journal editors' endorsement of CONSORT. As might be expected journals which endorsed CONSORT were more likely to respond (61%) to the survey than those journals which did not (39%). However, despite this limitation the results of this survey are still of interest, in particular, to those journals who said they would require, or recommend, authors comply with the CONSORT Statement but do not mention this in their 'Instructions to Authors'. This is particularly apparent for CONSORT extensions. In response to the survey two journal editors said that they were in the process of updating their journals' 'Instructions to Authors'. It is possible that these instructions were updated during the study period; however, the numbers are very small and would not affect our results. We note however, that there was inconsistency between editors' responses and the information on the journal's website under 'Instructions to Authors', especially in relation to the support of extensions to CONSORT.

Similar to the CONSORT Statement for reporting the results of randomized trials, other reporting guidelines exist which provide advice on how to report research methods and findings for other types of study designs. In our study journals which endorsed the CONSORT Statement were more likely to endorse other reporting guidelines, although the numbers were very low. The EQUATOR Network [14] is a new initiative which aims to increase awareness of good reporting guidelines in health research and thus improve the quality of scientific publications. We hope the work of this initiative will lead to better endorsement by journals of these important reporting guidelines.

## Conclusion

In summary, we believe that the CONSORT Statement, and its related extensions papers, will only continue to improve the quality of reports of randomized trials, if more journals endorse this initiative and, most importantly, require authors to comply as a condition of publication. The most obvious route through which this could be achieved is for journals to incorporate the CONSORT checklist and flow diagram into their editorial and peer-review processes and reflect this requirement in their published 'Instructions to Authors'. Without wide endorsement of the CONSORT Statement it cannot fully yield the benefits for which it was intended.

## Competing interests

All authors are involved in many initiatives in health care and healthcare research which should benefit from a wide uptake of the CONSORT Statement and related extensions. DGA, DM and KFS form the CONSORT Executive. SH is funded by a grant to support the work of the CONSORT Group.

## Authors' contributions

SH was involved in the design, implementation, analysis of the study, and in writing and commenting on drafts on the final manuscript. DGA, DM and KFS were involved in the design of the study and commenting on drafts on the final manuscript.
